# Predictors of arthritis in pediatric patients with lupus

**DOI:** 10.1186/s12969-015-0027-7

**Published:** 2015-07-14

**Authors:** SD Sule, DG Moodalbail, J Burnham, B Fivush, SL Furth

**Affiliations:** Johns Hopkins University, Baltimore, MD USA; Nemours/ Alfred. I. duPont Hospital for Children, Wilmington, DE USA; Department of Pediatrics, Perelman School of Medicine at the University of Pennsylvania and the Children’s Hospital of Philadelphia, Philadelphia, PA USA; Department of Epidemiology and Biostatistics, Perelman School of Medicine at the University of Pennsylvania, Philadelphia, PA USA

## Abstract

**Background:**

Arthritis is one of the most common manifestations of systemic lupus erythematosus (SLE). Although typically non-erosive and non-deforming, children with SLE arthritis can have significant morbidity with decreased quality of life. Our goal was to identify potential clinical and laboratory predictors of arthritis in a cohort of pediatric patients with SLE.

**Methods:**

We performed a cohort study of incident and prevalent patients with SLE aged ≤ 19 years. In cross sectional analysis, we compared demographic and clinical characteristics at initial clinic presentation between patients with arthritis noted at any time during follow-up and those without arthritis. We performed time to event analysis using Cox proportional hazard ratios to identify predictors of arthritis, clustering for repeated measures.

**Results:**

Forty seven children and adolescents with SLE were followed in the cohort, 91 % female and 68 % Black. In cross-sectional analyses, presence of malar rash was associated with arthritis. In longitudinal analyses, controlling for gender and race, increased age (HR: 1.4, 95 % CI: 1.1–1.7), malar rash (HR: 2.1, 95 % CI: 1.1–3.6), and presence of RNP antibodies (HR: 1.9, 95 % CI: 1.1–3.4) were predictive of arthritis. When controlling for gender, race, and medication use, anemia (HR: 8.5, 95 % CI: 2.9–24.2) and thrombocytopenia (HR: 6.1, 95 % CI: 2.4–15.6) were associated with increased risk of arthritis.

**Conclusions:**

We identified markers predictive of arthritis in a longitudinal cohort of children with SLE. The recognition of these markers may help clinicians identify patients at risk for arthritis before its onset thus improving quality of life in children with SLE.

## Background

Systemic lupus erythematosus (SLE) is a multi-system autoimmune disease that can present in childhood. Up to 20 % of patients with SLE are diagnosed in the pediatric population and females are more commonly affected than males [1].

Arthritis is one the most common manifestations of SLE in both children and adults and is in fact one of the criteria used to make the diagnosis of SLE [2–5]. The arthritis of SLE is typically non-erosive and non-deforming and involves both large and small joints [2].

Studies have shown that although the arthritis in SLE does not usually cause joint damage, it can be associated with significant decrease in quality of life in these children [6, 7]. In a cohort of 98 children with SLE, the presence of arthritis was associated with significantly worse quality of life and decreased function [6]. In comparison, other manifestations of SLE, including active lupus nephritis and renal damage, were not associated with decreased health-related quality of life. This indicates that although the arthritis of SLE is typically non-deforming, it is associated with significant morbidity in this population [6, 7]. Our goal was to define predictors of arthritis in a longitudinal cohort of pediatric patients with SLE.

## Methods

### SLE cohort

Incident and prevalent patients with SLE onset at age ≤19 years were recruited into this cohort study. These subjects were seen during their routine clinic visits every 3 months or more frequently if medically indicated. Subjects were recruited from Johns Hopkins University and the Children’s Hospital of Philadelphia in a longitudinal study from July 2008 through December 2012. Institutional review board approval was obtained at both institutions and all subjects/families enrolled in the cohort study consented for participation. For prevalent SLE patients, we performed retrospective chart review to collect clinical and laboratory data from the time of SLE diagnosis to cohort entry. All patients met the American College of Rheumatology (ACR) classification criteria for SLE [3–5].

At entry into the cohort, demographic data including age at visit, age at lupus diagnosis, gender, race, and ethnicity were collected. At each visit, history and physical exam data, medication, and laboratory information were collected. Laboratory data included complete blood count, erythrocyte sedimentation rate, basic metabolic profile, urinalysis, spot urine protein to creatinine ratio, complement levels (C3, C4), and autoantibodies including ANA, dsDNA, Ro, La, Smith (Sm), and RNP antibodies. The SLE Disease Activity Index (SLEDAI) and the Systemic Lupus Activity Measure (SLAM) disease activity measures were also calculated at each visit [8–12]. The SLEDAI is a weighted, cumulative index that is grouped into 9 organ systems. The organ systems include central nervous system, vascular, renal, musculoskeletal, serosal, dermal, immunologic, constitutional, and hematologic [11]. The SLAM is used to assess degree of disease activity within the preceding month and includes clinical manifestations and 8 laboratory parameters, each weighted [9].

### Definition of arthritis

Arthritis was based on the ACR definition in the classification criteria for SLE [3, 4]. This includes non-erosive arthritis involving two or more peripheral joints, characterized by tenderness, swelling, or effusion. To be included in the analyses, the arthritis must have been documented by the treating physician and have been present for at least the two weeks preceding the clinic visit.

### Statistical analysis

We performed *t*-test for comparison of means and chi-2 analyses for comparison of groups, comparing baseline clinical and demographic characteristics of patients without arthritis vs. those with arthritis at any time during cohort follow-up.

In longitudinal analyses, times to event calculations were used to determine the association between clinical, laboratory, and disease activity variables with arthritis. Time at risk was calculated using date of SLE diagnosis as the start date and either end of follow-up or December 31, 2012, the last date of data collected, as the end date. Failure, or the event of interest, was defined as the development of arthritis.

Cox proportional hazard ratios were used to determine predictors of arthritis. Both univariate and multivariate analyses were performed. In multivariate analyses, we controlled for potential confounding of race and gender. When analyzing the potential relationship between hematologic disease and arthritis, we controlled for race, gender, and immunosuppression use. Clustering by identification number was used to account for multiple measures within the same patient. If a subject presented with arthritis at the initial diagnosis of SLE, that visit was excluded from the longitudinal analyses since no prior data were available.

Demographic characteristics included age, gender and race. Laboratory data used in these analyses included autoantibodies including dsDNA, Ro, La, Smith, RNP, serum complement levels, hemoglobin, white blood cell count, platelet count, sedimentation rate, and C-reactive protein. Laboratory variables were defined as abnormal if they were noted to be outside of the recommended ranges. dsDNA titers were noted as either positive or negative; C3 was defined as low if <79 milligrams (mg) /deciliter (dl); C4 was low if < 12 mg/dl; low hemoglobin was defined as <11 grams (g)/dl, leukopenia as leukocyte count of <3000/mm^3^, thrombocytopenia as fewer than 100,000 platelets, sedimentation rate elevated if > 20 mm/h, and C-reactive protein was elevated if >0.5 mg/dl. Mean SLEDAI and SLAM disease activity scores were also included in the analyses. Missing data were imputed based on prior laboratory results. Other clinical findings of SLE were also included in the analyses including evidence of mucocutaneous involvement (malar rash, oral ulcers, alopecia), hematologic abnormalities, and kidney disease (defined as any type of lupus nephritis on kidney biopsy). There were too few patients with discoid rash alone (*n* = 1), neurologic (*n* = 1), or serosal involvement (*n* = 2) to include these SLE findings in the analyses. Immunosuppressive medication use at the visit prior to the development of arthritis was also included in the analyses. Data were analyzed using STATA, version 11 (Stata Corporation, College Station, TX). *P*-values less than 0.05 were considered significant.

## Results

47 pediatric patients with SLE were recruited into this cohort study with 126.5 person-years of longitudinal data. 90 % of patients were enrolled in the cohort at the time of initial diagnosis of SLE. In the remaining 10 % of subjects, data from the time of SLE diagnosis to time of cohort enrollment were collected through chart review. Demographic and clinical characteristics of subjects at the time of initial SLE diagnosis are shown in Table 1. Patients were divided into two groups: those without arthritis and those with arthritis at any time during longitudinal follow-up. There was no significant difference in baseline characteristics, autoantibody status, or disease activity scores between these groups. Malar rash was noted at the first clinic visit more often in patients who subsequently developed arthritis (50 % in those with arthritis vs. 16 % without arthritis, *p* = 0.02).

**Table 1:** Characteristics of Cohort at Initial Diagnosis of SLE

	No Arthritis during follow-up	Arthritis present during follow-up	*P*-value
	*N* = 25	*N* = 22	
Age, years (SD)	12.5 (3.5)	11.8 (4.2)	0.6
Gender, n (%) F	22 (88)	20 (90)	0.9
Race, % AA	17 (68)	14 (64)	0.3
ANA, n (%) positive	25 (100)	22 (100)	-
dsDNA, n (%) positive	21 (84)	15 (68)	0.4
Ro, n (%) positive	7/18 (39)	1/6 (16)	0.4
La, n (%) positive	5/18 (28)	0/6 (0)	0.1
RNP, n (%) positive	9/20 (45)	5/9 (55)	0.5
Sm, n (%) positive	11/20 (55)	4/8 (50)	0.4
Low C3, n (%)	21 (84)	13 (59)	0.8
Low C4, n (%)	15 (60)	13 (59)	0.8
Elevated CRP, n (%)	13 (52)	2 (9)	0.02
Elevated ESR, n (%)	18 (72)	13 (59)	0.4
Renal disorder, n (%)	16 (64)	9 (41)	0.8
Hematologic disorder, n (%)	11 (44)	4 (18)	0.2
Fever, n (%)	4 (16)	4 (18)	0.4
Malar rash, n (%)	4 (16)	11 (50)	0.02
Mucosal ulcerations, n (%)	2 (8)	2 (9)	0.6
Alopecia, n (%)	22 (88)	2 (9)	0.007
Mean SLEDAI	12.8 ± 14.2	10.7 ± 11.7	0.6
Mean SLAM	5.1 ± 3.6	5.4 ± 3.1	0.6

Laboratory values, SLEDAI, and SLAM scores were taken from the first presentation to the clinic. Renal, hematologic, skin, and integument involvement were defined as present if noted at any time during longitudinal follow-up

**Fig. 1:**
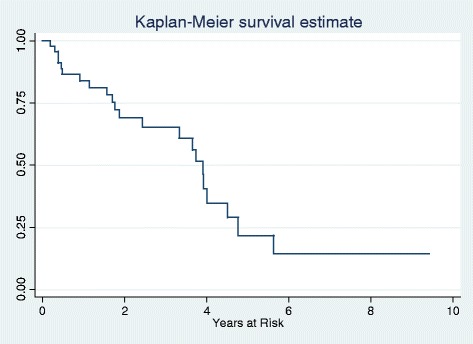
Timeline of Arthritis Development. Arthritis presented in all patients within two years of the diagnosis of SLE

In this cohort, 10/47 (21 %) had arthritis noted at multiple non-consecutive clinic visits for a total of 60 episodes of arthritis during longitudinal follow-up. 2/10 patients had arthritis at the initial diagnosis of SLE; no patients had only a single episode of arthritis. As show in Fig. 1, the majority of patients presented with arthritis within two years from time of SLE diagnosis. The most common joints involved included the hand (8/10), wrist (6/10), knee (4/10), and elbow (3/10). None of the patients had radiologic evaluation.

We next sought to identify potential predictors of arthritis over time in the cohort (Table 2). In univariate analyses, increasing age was associated with arthritis (Hazard Ratio (HR): 1.3, 95 % Confidence Interval (CI): 1.1–1.6). Patients with antibodies to RNP were at increased risk of developing arthritis (HR: 2.1, 95 % CI: 1.2–3.5). Arthritis was less likely in patients of Black race compared to Caucasians.

**Table 2:** Risk of arthritis in longitudinal analyses

	Cox proportional hazard ratio	Cox proportional hazard ratio
	Univariate (95 % Confidence interval)	Controlling for race and gender (95 % Confidence interval)
Age, years	1.3 (1.1–1.6)	1.4 (1.1–1.7)
Male gender	2.9 (0.8–9.7)	1.6^a^ (0.4–6.3)
Black race	0.3 (0.1–0.9)	0.3^b^ (0.1–1.1)
dsDNA	1.5 (0.5–4.3)	1.5 (0.5–4.2)
Ro	0.6 (0.3–1.6)	0.8 (0.3–2.2)
La	1.3 (0.3–5.4)	1.4 (0.3–6.6)
RNP	2.1 (1.2–3.5)	1.9 (1.1–3.4)
Sm	0.6 (0.2–1.8)	0.3 (0.06–1.3)
Low C3	0.6 (0.3–1.1)	0.6 (0.3–1.2)
Low C4	0.7 (0.3–1.6)	0.7 (0.3–1.5)
Elevated CRP	0.9 (0.8–1.1)	0.9 (0.8–1.1)
Elevated ESR	0.5 (0.3–1.1)	0.6 (0.3–1.3)
Renal disorder	1.1 (0.5–2.9)	1.2 (0.4–2.8)
Anemia	8.8 (2.7–28.5)	8.5^c^ (2.9–24.2)
Thrombocytopenia	3.1 (0.8–11.7)	6.1^c^ (2.4–15.6)
Leukopenia	1.1 (0.3–4.5)	0.8^c^ (0.2–2.9)
Fever	1.2 (0.4–3.9)	1.3 (0.3–5.4)
Malar rash	1.8 (0.9–3.2)	2.1 (1.1–3.6)
Mucosal ulcerations	1.6 (0.8–3.1)	1.3 (0.6–2.9)
Alopecia	0.8 (0.4–1.5)	0.8 (0.4–1.5)
Prednisone use	0.4 (0.2–3.6)	0.9 (0.7–3.2)
Immunosuppressive medication use	0.8 (0.5–1.4)	1.05 (0.6–1.7)
SLEDAI score	1.1 (0.9–1.2)	0.9 (0.8–1.1)
SLAM score	1.1 (0.9–1.3)	1.1 (0.8–1.4)

^a^Controlling for Race only; ^b^Controlling for Gender only; ^c^Controlling for Race, Gender, and Immunosuppression use

When controlling for race and gender, increasing age remained a significant predictor of arthritis (HR: 1.4, 95 % CI: 1.1–1.7). The presence of RNP antibodies also remained predictive of arthritis. Those with a malar rash had a two-fold increased risk of arthritis compared to patients with no skin involvement (HR: 2.1, 95 % CI: 1.1–3.6). Anemia was predictive of arthritis, even after controlling for use of immune suppression, race, and gender (HR: 8.5, 95 % CI: 2.9–24.2). Patients with thromobocytopenia had a six-fold increased risk of arthritis, even after controlling for potential confounders (HR: 6.1, 95 % CI: 2.4–15.6).

Non-steroidal anti-inflammatory agents were used as the initial treatment for arthritis in all of the patients. In two patients, a steroid tapering dose pack was also used as treatment for arthritis. Methotrexate was started in three of the patients at the visit where arthritis was recorded. In longitudinal analyses, there was no association between development of arthritis and use of immunosuppressive medication at the visit prior (HR: 0.8, 95 % CI: 0.5–1.4).

## Discussion

In this longitudinal study, we found that approximately 20 % of patients had at least one episode of non-deforming arthritis during follow-up. The majority of patients presented with arthritis within two years of the diagnosis of SLE. Older, Caucasian children were at increased risk of developing arthritis. Both the presence of anti-RNP antibodies and malar rash doubled the risk of developing arthritis in this cohort. Hematologic abnormalities, including anemia and thrombocytopenia, were predictive of future arthritis.

Joint symptoms, including arthritis and arthralgia, have been noted in 20-90 % of patients with childhood-onset SLE [13]. Saketoo et al. classified lupus arthritis into five categories: 1) SLE arthralgia without inflammatory signs such as erosions or deformity; 2) non-deforming, non-erosive inflammatory arthropathy; 3) non-deforming, erosive arthropathy; 4) mechanically erosive deforming arthropathy; and 5) synovial erosive deforming arthorpathy [14]. Others such as Ball et al. have divided lupus arthritis into three broad categories: 1) deforming, non-erosive arthopathy including Jacoud’s arthropathy; 2) mild deforming arthropathy which includes patient who have obvious deformity and no erosions; and 3) erosive arthritis [15].

The most common type of lupus arthritis is non-deforming, non-erosive joint inflammation [15]. This differs from the arthritis seen in children with juvenile idiopathic arthritis, which is typically inflammatory and can lead to significant joint damage and erosions. SLE arthritis most commonly involves the small joints of the hands and feet as well as larger joints such as knees, ankles, wrists. Chronic arthritis, defined as persistence of arthritis for more than 6 weeks, is rare in childhood SLE with a prevalence of 2.6 % [16]. None of the children in our cohort had arthritis for more than 4 consecutive weeks.

In a cross-sectional analysis of 533 adult patients with SLE, arthralgias and arthritis were noted in 83 % of the cohort [17]. When comparing patients with articular involvement vs. without articular involvement, it was noted that female gender, fevers, oral ulcers, and alopecia were more prevalent in the group with articular disease [17]. In our population, these factors were not associated with arthritis. In fact, children without arthritis were noted to have a statistically significant increase in alopecia at initial clinic presentation. These differences in SLE disease associations with arthritis may be due to variations between childhood and adult SLE.

In a study of 1,082 Chinese patients with SLE, distinct clusters of SLE disease were noted [18]. The first cluster was noted to have relatively benign disease with skin involvement, arthritis, and photosensitivity. The second group was noted to have serositis, hematologic involvement, and renal disease. The third subset was noted to have arthritis, hematologic involvement, and renal disease. Although the latter two groups had similar renal and hematologic presentations, the group with these manifestations plus arthritis had a significantly better prognosis. The authors felt that the presence of skin and joint disease may be associated with improved outcomes in patients with SLE. It was also noted that the first cluster of patients, with predominately skin and joint involvement, had relatively benign disease with very few flares. This benign clinical subset of SLE patients has been reported by other investigators as well [19, 20].

In this study, we found that children with SLE and RNP antibodies were at increased risk of developing arthritis. In a comparison of adult SLE patients with deforming, non-erosive arthritis, it was noted that there was association between presence of arthropathy and anti-dsDNA antibodies [21]. In a separate study of thirteen patients with SLE and deforming arthritis, a higher prevalence of anti-Ro and anti-La antibodies were noted compared to those without arthritis [22]. We did not routinely check rheumatoid factor or anti-cyclic citrullinated peptide (CCP) antibodies in this cohort. Other investigators have noted an increased risk of erosive arthritis in adult SLE patients who have concomitant anti-CCP antibodies [21, 23–26].

Anemia and thrombocytopenia were predictors of arthritis in our cohort. In a study by Cavalcante et al. of seven pediatric patients with SLE and chronic polyarthritis, hematologic involvement was noted in six of the seven subjects [16]. Thrombocytopenia has been associated with worse prognosis in adults with SLE [27]. The presence of thrombocytopenia significantly worsened the probably of survival over 15 years in a cohort of 389 adults with SLE. It is possible that these hematologic findings of anemia and thrombocytopenia are surrogates for worse disease.

Our study has some limitations. We combined all immunosuppressive medications, including hydroxychloroquine, methotrexate, mycophenolate mofetil, and cyclophosphamide into one variable because medication use varied throughout the cohort and differences were noted in prescribing practice and treatment of SLE between the two centers. Other disease manifestations may prompt earlier immune suppression treatment and alter the expression of arthritis. Additionally, we did not routinely check antibodies more commonly associated with chronic arthritis such as rheumatoid factor or anti-CCP antibodies. Strengths of our study include the longitudinal data collection with the same treating physician at each site performing the physical exams.

## Conclusions

Identifying risk factors for arthritis is important because of the negative impact of joint pain on quality of life and function in these children. In this study, we have shown that arthritis is relatively common in a cohort of pediatric patients with SLE. Predictors of arthritis include increasing age, malar rash, autoantibodies, and hematologic disorder. The recognition of these potential predictors may help clinicians identify patients at risk for arthritis before its onset.

This work was supported by a National Institutes of Health [5K23AR052736 to S.Sule] and by generous donations from the Hock and Mangione families.
